# Supplementary material for: What are the modifiable factors of treatment burden and capacity among people with Parkinson’s disease and their caregivers: A qualitative study
Source: PLoS One. 2023 Mar 30;18(3):e0283713. doi: 10.1371/journal.pone.0283713 (PMC10062656; doi:10.1371/journal.pone.0283713)
Supplement: S1 File — (PDF) [file pone.0283713.s001.pdf]

S1 File: INTERVIEW GUIDES:

Exploring the treatment burden and capacity of people with Parkinson's

Introduction

1. Give complete name.
2. Introduce self as researcher from Academic Geriatric Medicine at the University of Southampton.
3. Give short explanation about the study and purpose of the interview.
4. Discuss confidentiality and confirm consent for audio recording.

- *I would like to understand your experiences of looking after your health and living with Parkinson's. I want to find out your views on how you have learnt to live and deal with your condition, the treatments and advice you have been given.*
- *There are no right and wrong answers, whatever you say will be helpful and allow us to get a better understanding of the issues. Please let me know if you find any issues distressing or if you do not wish to answer any question. If at any time you want to stop, or have a break, please feel free to let me know.*
- *Everything you tell me today will be kept confidential. I will be recording the interview, so I can remember all you have said to me.*
- *I would like to start by asking some generic questions about your Parkinson's.*

General Questions about Parkinson's

1. How long have you been diagnosed with Parkinson's?
   How did you get diagnosed? Who did you see? How long did it take?
2. How did you get information about Parkinson's?
   How easy or difficult was it obtaining the information?
   How easy or difficult do you find understanding the information about Parkinson's?
3. Do you have other medical conditions? What are they?
   Which of your medical conditions including Parkinson's do you consider to be the main issue at this moment?
4. What has the impact of Parkinson's been on your life or social networks?
5. Do you feel overstretched with everything you need to do for your Parkinson's?

Treatment Burden in Parkinson's

Thinking about your Parkinson's…

6. Are there aspects of looking after your Parkinson's that you find difficult of challenging?
   What are they?

<u>The following topics will be explored in an open fashion if appropriate, building on the response from participants to Question 6:</u>

a. Do you **monitor** your Parkinson's? What do you do to monitor your Parkinson's? What aspects do you find difficult or challenging?<br>b. How do you manage your **medications and prescriptions**? What do you do? Are there any challenges with this? How many treatments/medications do you take?<br>c. Have you had any **financial** expenses associated with managing your Parkinson's? What was that for? Was there any help with that?<br>d. How many times have you seen your **GP** about your Parkinson's in the last 12 months? Tell me about your experiences at the GP. Can you tell me about your experiences with your GP before COVID?<br>e. How many **hospital appointments** have you had for your Parkinson's in the last 12 months? Tell me about your experiences at the hospital appointment. Can you tell me about your experiences with hospital appointments before COVID?<br>f. Have you had contact or access to **other healthcare services** (district nurses, physiotherapist, occupational therapist, speech therapist etc.)? How do you find attending all the appointments?<br>g. Have you had any issues **getting help at the weekends or overnight**? How did you find this experience?

7. What do you do if you have a **concern or question** about your Parkinson's?
Tell me about your experiences of getting hold of your Parkinson's doctors/GP/nurse specialist/therapist. Was this different before COVID?

8. What are your thoughts about how your **care is coordinated** between all the health care professionals such as your GP, hospital doctors, therapists or nurses?
Have you had to do anything to resolve any issues or miscommunication?

Capacity

9. What things have helped you deal with the issues you have mentioned earlier such as…? *(remind them of the issues that was mentioned from previous questions)*

10. Do you have help from anyone such as a family or friend to help with your Parkinson's?

11. Do you have any paid carers that help you with your personal care?
If yes - how easy or difficult was to organise this? If no – why not?
Are you aware of where to get information about getting more help if needed?

12. How can the healthcare system could be changed to help you manage your health with Parkinson's?

13. Is there anything else you like to add?

Interview Details

Subject ID:	
Date of Interview:	
Time of Interview:	
Interviewer:	

Participant Profile

1. Age:

2. Gender: ☐ Male ☐ Female

3. Relationship of caregiver (if they have one):

4. Living arrangements: ☐ Alone
☐ With Spouse/partner/family/friend
☐ Other: ____________________

5. Ethnicity: ☐ White
☐ Asian/Asian British
☐ Black /African/Caribbean/Black British
☐ Mixed/Multiple ethnic groups
☐ Other (please specify) ________________

6. Can you tell me if you went onto any further education after secondary school?

☐ No ☐ Yes (please describe: _____________________________)

7. How does your Parkinson's affect you at present? (tick one box)
☐ No sign of disease
☐ Parkinson's symptoms on one side of the body
☐ Parkinson's symptoms on both sides of the body with no balance problems
☐ Mild to moderate Parkinson's symptoms on both sides of the body with some balance problems but still physically independent
☐ Severe disability but still able to walk or stand unassisted
☐ Wheelchair-bound or bedridden unless assisted

Close of the interview

Thank you for your time and participation. I would like to reassure you that everything you told me will be kept confidential and identified only by an identity number.

Exploring the treatment burden and capacity of caregivers of people with Parkinson's

Introduction

5. Give complete name.
6. Introduce self as researcher from Academic Geriatric Medicine at the University of Southampton.
7. Give short explanation about the study and purpose of the interview.
8. Discuss confidentiality and confirm consent for recording.

- *I would like to understand your experiences of helping to care and support the health of someone living with Parkinson's. I want to find out your views on how you have learnt to live and deal with their health with Parkinson's, the treatments and advice you have been given.*
- *There are no right and wrong answers, whatever you say will be helpful and allow us to get a better understanding of the issues. Please let me know if you find any issues distressing or if you do not wish to answer any question. If at any time you want to stop, or have a break, please feel free to let me know.*
- *Everything you tell me today will be kept confidential. I will be recording the interview, so I can remember all you have said to me.*
- *I would like to start by asking some general questions about (name of person), who has Parkinson's.*

General Questions about Parkinson's and health

1. How long have they been diagnosed with Parkinson's?
   How did they get diagnosed? Who did you see? How long did it take?
2. How did you get information about Parkinson's?
   How easy or difficult was it obtaining the information?
   How easy or difficult do you find understanding the information about Parkinson's?
3. Do they have other medical condition? What are they?
   Which of the medical conditions including Parkinson's do you consider to be the main issue at this moment?
4. What is your role in helping to support or care for *(name of person)?*
   Has this changed over time – *if yes, in what way?*
5. What is the impact of your caring role on your life or social networks?
6. Do you feel overstretched with everything you have to do to help their Parkinson's?

Treatment Burden in Parkinson's

Thinking about (name of person with Parkinson's)…

7. Are there aspects of looking after their Parkinson’s that you find difficult or challenging? What are they?

<u>The following topics will be explored in an open fashion if appropriate, building on the response from participants to Question 6:</u>

a. Do you **monitor** their Parkinson’s? What do you do to monitor their Parkinson’s? What aspects do you find difficult or challenging?

b. Do you help them with **medications and prescriptions**? What do you do? Are there any challenges with this? How many treatments/medications do they have?

c. Have you had any **financial** expenses associated with managing their Parkinson’s? What was that for? Was there any help with that?

d. How many times have you seen **the GP** about their Parkinson’s in the last 12 months? Tell me about your experiences with the GP. Can you tell me about your experiences with the GP before COVID?

e. How many **hospital appointments** have they had for their Parkinson’s in the last 12 months? Tell me about your experiences at the hospital appointment.
Can you tell me about your experiences with hospital appointments before COVID?

f. Have you had contact or access to **other healthcare services** (district nurses, physiotherapist, occupational therapist, speech therapist etc.)?
How do you find attending all the appointments?

g. Have you had any issues **getting help at the weekends or overnight**?
How did you find this experience?

8. What do you do if you have a **concern or question** about their Parkinson’s?
Tell me about your experiences of getting hold of your Parkinson’s doctor/GP/nurse specialist/therapist. Was this different before COVID?

9. What are your thoughts about how your **care is coordinated** between all the health care professionals such as your GP, hospital doctors, therapists or specialist nurses?
Have you had to do anything to resolve any issues or miscommunication?

Capacity

10. What things have helped you deal with the issues you have mentioned earlier such as… ?
(remind them of issues that was mentioned from previous questions)

11. Does *(name of person with Parkinson’s)* have paid carers to help with their personal care?
If yes - how easy or difficult was to organise this? If no – why not?
Are you aware of where to get information about getting more help if needed?

12. How can the healthcare system be changed to help you manage their health with Parkinson’s?

General Questions About the Caregiver

What about yourself, do you have any medical conditions? What are they?

13. What do you do to look after your own health?
Does this affect your ability to look after *(name of the person with Parkinson's)?* Has helping to look after *(name of person with Parkinson's)* affected your own health?

14. Is there anything else you like to add?

Interview Details

Subject ID:	
Date of Interview:	
Time of Interview:	
Interviewer:	

Participant Profile

1. Age:

2. Gender: ☐ Male ☐ Female

3. Relationship to person with Parkinson's:

4. Living arrangements: ☐ Alone
   ☐ With Spouse/partner/family/friend
   ☐ Other: ____________________

5. Ethnicity: ☐ White
   ☐ Asian/Asian British
   ☐ Black /African/Caribbean/Black British
   ☐ Mixed/Multiple ethnic groups
   ☐ Other (please specify) ________________

6. Can you tell me if you went onto any further education after secondary school?

   ☐ No ☐ Yes (please describe: ___________________________)

7. How does the Parkinson's affect the person with Parkinson's at present? (tick one box)
   ☐ No sign of disease
   ☐ Parkinson's symptoms on one side of the body
   ☐ Parkinson's symptoms on both sides of the body with no balance problems
   ☐ Mild to moderate Parkinson's symptoms on both sides of the body with some balance problems but still physically independent
   ☐ Severe disability but still able to walk or stand unassisted
   ☐ Wheelchair-bound or bedridden unless assisted

Close of the interview

Thank you for your time and participation. I would like to reassure you that everything you told me will be kept confidential and identified only by an identity number.
