# Supplementary material for: What are the modifiable factors of treatment burden and capacity among people with Parkinson’s disease and their caregivers: A qualitative study
Source: PLoS One. 2023 Mar 30;18(3):e0283713. doi: 10.1371/journal.pone.0283713 (PMC10062656; doi:10.1371/journal.pone.0283713)
Supplement: Attachment — Submitted filename: Response to Reviewers.docx [file pone.0283713.s002.docx]

**Rebuttal letter**

Dear Academic Editor,

Re: PONE-D-22-24429

Manuscript Title: *What are the modifiable factors of treatment burden and capacity among people with Parkinson’s disease and their caregivers: A qualitative study*

Thank you for taking your time to review our manuscript and for giving us the opportunity to respond to your comments and those of the reviewers. I would also like to thank the reviewers for their constructive feedback which we have worked towards and summarised the changes below. We hope this has helped enhanced the manuscript. The table on the next page depicts how the comments were addressed with corresponding tracked changes in the attached revised manuscript.

| **No** | **Reviewers’ comments** | **How the comments were addressed** | **Section/Page/Line of where comments were addressed** |
| --- | --- | --- | --- |
| **Academic Editor** | | | |
| 1. | Please ensure that your manuscript meets PLOS ONE's style requirements, including those for file naming. | Thank you for your comment. We have ensured the manuscript meets the PLOS ONE’s style requirements. | Whole document |
| **Reviewer #1** | | | |
| 2. | L 1 Title: The disorder should be Parkinson's disease, not just Parkinsons. Is it Parkinson's law, gait, syndrome, plus syndrome. A minor point , but needs review. | Thank you for your comment. For further clarification we have amended the title as suggested and ensured the term Parkinson’s disease is used throughout the article. | Title, Page 1, L2, L4 |
| 3. | L31 Abstract methods: Hoehn and yahr stages need a brief explanation | Thank you for your comment. We have included a brief explanation for Hoehn and Yahr to indicate the severity of Parkinson’s disease | Abstract, Page 2, L32 |
| 4. | L48: The term long term condition though validate would be better served by the use of chronic disease, which may explain a disorder with no cure relying on palliative care and medications. | Thank you for your comment. The terms ‘long-term condition’ and ‘chronic disease’ may be used interchangeably to depict a health condition with no cure. As such, we have decided to continue with the term long-term condition in this article. | No changes |
| 5. | L88: The abbreviation QYT has not been explained, confusing | Thank you for your comment. We have included a brief explanation that QYT indicates the 1^st^ author | Participant Recruitment and Sampling, Page 4, L92 |
| 6. | L96: S1 file: Not explained and no indication as to its location, is it an appendix? | Thank you for highlighting this. The S1 File has been uploaded onto PLOS ONE as per the editorial manuscript requirements for review. | No changes |
| 7. | L112: How were the participants anonymized, before data collection and analysis? Not stated | Thank you for your comment. As seen in L114, data were anonymised prior to data analysis. | No changes |
| 8. | L114: What is K1? Not clear | Thank you for your comment. We have added an explanation highlighting KI as the last author. | Data analysis, Page 5, L116 |
| 9. | L115: Was a specific qualitative framework employed for the inductive data coding and theme generation? Not clear | Thank you for your comment. Data analysis was conducted using thematic analysis, with Braun and Clarke 2006 cited accordingly. This method analysis does not employ the use of a qualitative framework. Rather, familiarisation of data and generation of initial codes systematically and identification of themes are conducted and then re-reviewed to develop final refinement of each theme and relationship between themes. | No changes |
| 10. | L119: What type of mind map? Diagram or figure would be appropriate, at least some form of explanation. | Thank you for your comment. We have briefly explained this further. | Data analysis, Page 6, L121 |
| 11. | L122: QYT only now explained, however, is the female clinician, medical, nursing, paramedical? Not clear | Thank you for your comment. We have clarified the role of QYT. | Reflexivity, Page 6, L125 |
| 12. | L129 Pwp, 8 caregivers, now introduced, not previously explained. | Thank you for your comment. We have now added this information into the earlier section ‘Participant Recruitment and Sampling’ | Participant Recruitment and Sampling, Page 4, L95 |
| 13. | L406: Strengths and limitations, purposive sampling should also have been mentioned in the sampling method and clarified as to what method employed. | Thank you for your comment. Purposive sampling was described in section ‘Participant Recruitment and Sampling’, page 4, L85 | No changes |
| **Reviewer #2** | | | |
| 14. | While other authors have described treatment burden and the ability to manage said burden, (termed capacity), in the context of PD, this group reports to be the first exploring this relationship in PWP and from care partners and proposes factors which may be modifiable. 9PWP and 8 partners were interviewed and they identified 4 themes related to treatment burden: 1) appointment and access challenges; 2) information gathering; 3) Managing prescriptions; 4) Personal lifestyle changes.  Areas relevant to capacity were numerous and included transportation, geography, health literacy, social support, computer literacy and access, and financial support, personal attributes (positive attitude, sense of humor), and faith. Unique to this work is identifying prescription errors, medication availability, collecting prescriptions, and issues with access to GP as novel factors contributing to treatment burden.  Any study that attempts to understand and improve quality in complex neurodegenerative conditions is laudable. It is also a logical extension of their previously cited work. | Thank you for your positive comments and highlighting the novel findings of treatment burden and capacity in Parkinson’s disease that this research study has identified. | No changes |
| 15. | My major comment is to consider re-working the discussion so it is more practical for a local PD specialist or even a clinic manager. As it stands, it reads mostly like a reiteration of the results section. Selected examples follow:  How should one approach frequency of healthcare appointments (line 348)? And how would you use the data gathered to support this since the participants seemed to offer conflicting reports: some wanted more and some wanted fewer visits with their specialists.  What about “…medication reviews to reduce polypharmacy may help reduce the treatment burden experienced” (line 352)? Should this be done with the specialist, the GP, a pharmacist working with the treatment team? What change in workflow can be implemented or what strategies can be employed to empower the PWP or dyad to address this?  What about improving health literacy (line 340)? HOW does one go about doing this, and should it fall on the neurologist?  What about maintaining positivity (line 356)? This is nice to encourage but depression and apathy are well-established features of PD, are probably under-diagnosed and under-treated, and should not be ignored.  While not necessary, the authors might consider putting together another figure/table which summarizes these findings in the way they do for the results. | Thank you for your comments. Your comments regarding the discussion section have been helpful in shaping the revised article. We have rewritten the second and third paragraph of the Discussion section in order to highlight the potential practical examples for change that may be implemented from the study findings.  Thank you for the suggestion for an additional figure or table. We gave this serious consideration and have added more to the discussion narrative in the article. | Discussion, Page 20-21, L347-375 |
| 16. | Furthermore, I believe the strengths are oversold. I appreciate that a wide spectrum of disease is represented, and there is considerable burden on analyzing qualitative data, but having one person with DBS is unlikely to represent the collective attitudes of the PD+DBS population. | Thank you for your comment. We have noted the small sample size for patients with DBS and PD dementia and included this as a limitation. | Strengths and Limitations, Page 23, L489-430 |
| 17. | The authors’ limitations are well-stated. “treatment burden amongst caregivers of people with long-term conditions remains understudied” (line 396) was an unfortunate missed opportunity. | Thank you for your comment. We agree that whilst this work has tried to fill the gap of knowledge around treatment burden amongst caregivers in PD, further research specifically focused on the caregiver treatment burden is required and has been recognised in a review by Sheehan et al (doi: [10.1186/s12877-019-1222-z](https://doi.org/10.1186%2Fs12877-019-1222-z)). | No changes |
| 18. | I do wonder if you would be able to look at zip code of the participants as a proxy for financial status? This may partially address one of the limitations mentioned. | Thank you for your comment. In the UK, post codes may be used as a proxy for deprivation status and average disposable income although it is a reflective of the neighbourhood rather than individual circumstances. We have therefore decided not to use this during data analysis. | No changes |
| 19. | The use of telemedicine in PD specifically has been done, and I point the authors to: Beck, C.A.; Beran, D.B.; Biglan, K.M.; Boyd, C.M.; Dorsey, E.R.; Schmidt, P.N.; Simone, R.; Willis, A.W.; Galifianakis, N.B.; Katz, M., et al. National randomized controlled trial of virtual house calls for Parkinson disease. Neurology 2017, 89, 1152-1161, doi:10.1212/wnl.0000000000004357. | Thank you for highlighting this important article. We have included this as a reference and amended the sentence accordingly. | Discussion, Page 22, L400-401 |
| 20. | And a trivial comment. Line 69: should read ‘…neurosurgical procedures SUCH AS deep brain stimulation’ | Thank you for your comment. We have amended the sentence as recommended. | Introduction, Page 3, L70 |
